# Highly Permeable Graphene Oxide/Polyelectrolytes Hybrid Thin Films for Enhanced CO_2_/N_2_ Separation Performance

**DOI:** 10.1038/s41598-017-00433-z

**Published:** 2017-03-28

**Authors:** Jiwoong Heo, Moonhyun Choi, Jungyun Chang, Dahye Ji, Sang Wook Kang, Jinkee Hong

**Affiliations:** 1 School of Chemical Engineering & Material Science, Chung-Ang University, 84 Heukseok-ro, Dongjak-gu, Seoul, 06974 Republic of Korea; 2 Department of Chemistry, Sangmyung University, Seoul, 03016 Republic of Korea

## Abstract

Separation of CO_2_ from other gasses offers environmental benefits since CO_2_ gas is the main contributor to global warming. Recently, graphene oxide (GO) based gas separation membranes are of interest due to their selective barrier properties. However, maintaining selectivity without sacrificing permeance is still challenging. Herein, we described the preparation and characterization of nanoscale GO membranes for CO_2_ separation with both high selectivity and permeance. The internal structure and thickness of the GO membranes were controlled by layer-by-layer (LbL) self-assembly. Polyelectrolyte layers are used as the supporting matrix and for facilitating CO_2_ transport. Enhanced gas separation was achieved by adjusting pH of the GO solutions and by varying the number of GO layers to provide a pathway for CO_2_ molecules. Separation performance strongly depends on the number of GO bilayers. The surfaces of the multilayered GO and polyelectrolyte films are characterized by atomic force microscopy and scanning electron microscopy. The (poly (diallyldimethylammonium chloride) (PDAC)/polystyrene sulfonate (PSS)) (GO/GO) multilayer membranes show a maximum CO_2_/N_2_ selectivity of 15.3 and a CO_2_ permeance of 1175.0 GPU. LbL-assembled GO membranes are shown to be effective candidates for CO_2_ separation based on their excellent CO_2_/N_2_ separation performance.

## Introduction

Separation of CO_2_ from gas mixtures is of interest due to its corrosive nature in industrial environments, such as pipelines, and as a means of reducing greenhouse gasses that contribute to global warming^1, 2^. Among the separation method, membrane-based separation is widely used because of simple operation and low cost^3^. Research on CO_2_ separation has included investigation of a variety of polymeric membranes^4, 5^, inorganic membranes^6^, carbon membranes^7^, alumina membranes^7^, zeolite membranes^8^ and hybrid membranes and inorganic membranes^9^. Polymeric membrane was studied by various groups; Illing *et al*. reported polyaniline based membranes and the highest selectivity for CO_2_/N_2_: 17.0^5^ and Iqbal *et al*. reported asymmetric polycarbonate membranes with maximum ideal selectivity for CO_2_/CH_4_: 173.88^10^. Many researches on hybrid membranes and inorganic membranes were studied such as, polyimide-carbon molecular sieve mixed membrane^11^, asymmetric nafion/(zirconium oxide) hybrid membrane^12^ and Mixed matrix hollow fiber membranes made with modified HSSZ-13 zeolite in polyetherimide polymer matrix^13^.

Among the various separation methods, membrane techniques utilizing graphene oxide (GO) show great potential in CO_2_ separation. GO is a well-known two-dimensional chemical compound based on a partially allotropic form of carbon. Although GO is one-atom thick, it has unique electrical properties and can serve as a perfect barrier to ions and molecules. The electron cloud of the GO π-orbitals blocks even the smallest gas molecules^14^. Recent research has revealed that GO can function as a gas separation membrane when its pore and stacking structures are controlled^15^. Park *et al*. reported selective gas transport through a few-layer GO membrane by adjusting centrifugal force and electrostatic repulsion during the adsorption process of GO sheets^16^. Also, Xu *et al*. have reported selective membranes with gas transport channels formed from laminar GO^17^.

Until now, GO membranes have been prepared mostly in aqueous solution and processed using spin coating, drop casting, and filtration methods. However, these GO membranes present several challenges. For example, the previously reported coating methods make difficulties in precise control of thickness and stacking density. Furthermore, GO membranes without a supporting layer are brittle, which is a limitation in industrial applications. In addition, electrostatic repulsion arising from the carboxyl groups in GO causes undesirable cracks that lead to low selectivity.

Herein, we propose a spray-assisted layer-by-layer (LbL) self-assembly method for precisely controlling the preparation of GO sheets to be used as CO_2_ separation membranes. LbL assembly is a well-developed multilayer deposition process. The most important advantage of the method is that it offers precise control of film thickness and internal structure without limitation by substrates and materials through complementary interactions (i.e., electrostatic interactions, covalent interactions, and hydrogen bonding)^18–24^.

To take full advantage of LbL assembly, we fabricated GO membranes that enhance two components of the CO_2_ separation process. The first component involves the strong CO_2_ affinity of the numerous polar groups on GO and the molecular sieving effect of aligned GO layers. The second component is the facilitation of CO_2_ transport by amine groups in the polyelectrolyte layers. However, conventional dipping LbL assembly is not suitable for preparation of membrane substrates, because the flexibility of polysulfone (PSf) leads to destruction of deposited nanoscale films. Therefore, we adopted a spray method to deposit a stable nanofilm on the flexible substrate^25^.

## Results and Discussion

**Figure 1:**
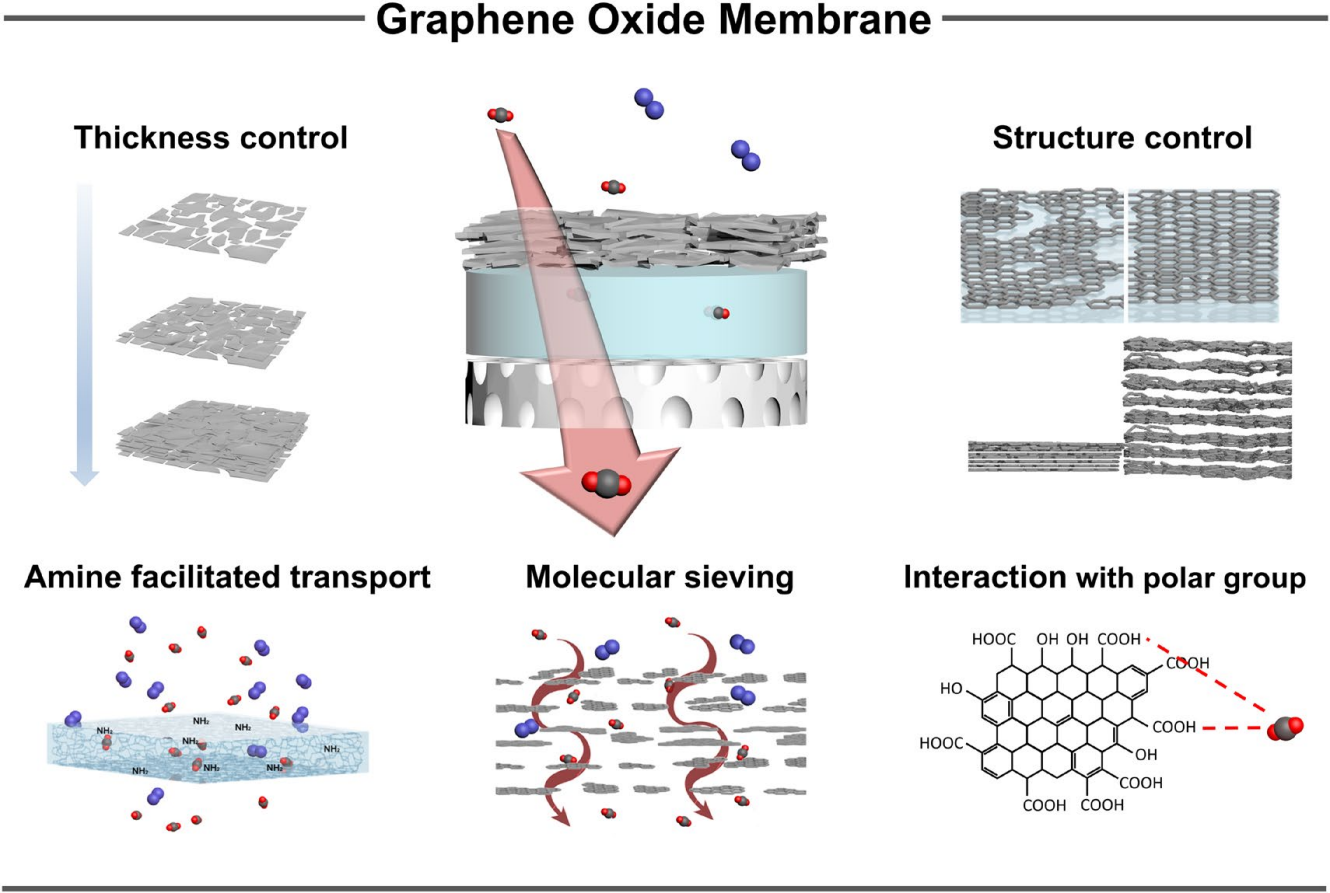
Schematic illustration of LbL-assembled polyelectrolyte/GO membranes for CO_2_ separation.

According to previous research, the CO_2_ separation performance of GO membranes results from the interlayer spaces in stacked GO sheets^26–28^. Defective pores were generated during both the oxidation and ultrasonication processes^29^. Ultrasonication is used to promote the dispersion of the GO sheet in aqueous solution. Porous support membrane can be covered with an increasing number of GO layers, which increases the CO_2_ selectivity. Conversely, CO_2_ permeance declines as the thickness of the GO layers increases because of the longer diffusion pathway that CO_2_ molecules must traverse to pass through the membrane. The balance between selectivity and permeance is extremely important in CO_2_ separation membranes, and it can be achieved by the precise control of the stacking structure of GO sheets. Therefore, we designed a GO membrane as shown in Fig. 1. The supporting matrix of the repeating (PDAC/PSS)_n_ layers was first deposited onto the porous PSf surface, and the (GO/GO)_n_ layers were then introduced selectively. The LbL film notation used here is (PDAC/PSS)_n_, representing a multilayer film consisting of PDAC and PSS, where n is the number of bilayers. For example, (PDAC/PSS)_25_ represents a 25-bilayer multilayer film consisting of repeated PDAC/PSS layers. Although the intrinsic barrier properties of GO layers block N_2_ molecules, a certain number permeate through non-selective cracks. The role of LbL-assembled polyelectrolyte multilayer is preventing the undesirable permeation of N_2_ molecules through cracks and wrinkles in the GO layers, and to enhance selectivity for CO_2_ over N_2_. Furthermore, a (PDAC/PSS)_n_ layer covers the pores of the PSf membrane and prevents disordered GO aggregation in the membrane pores.

**Figure 2:**
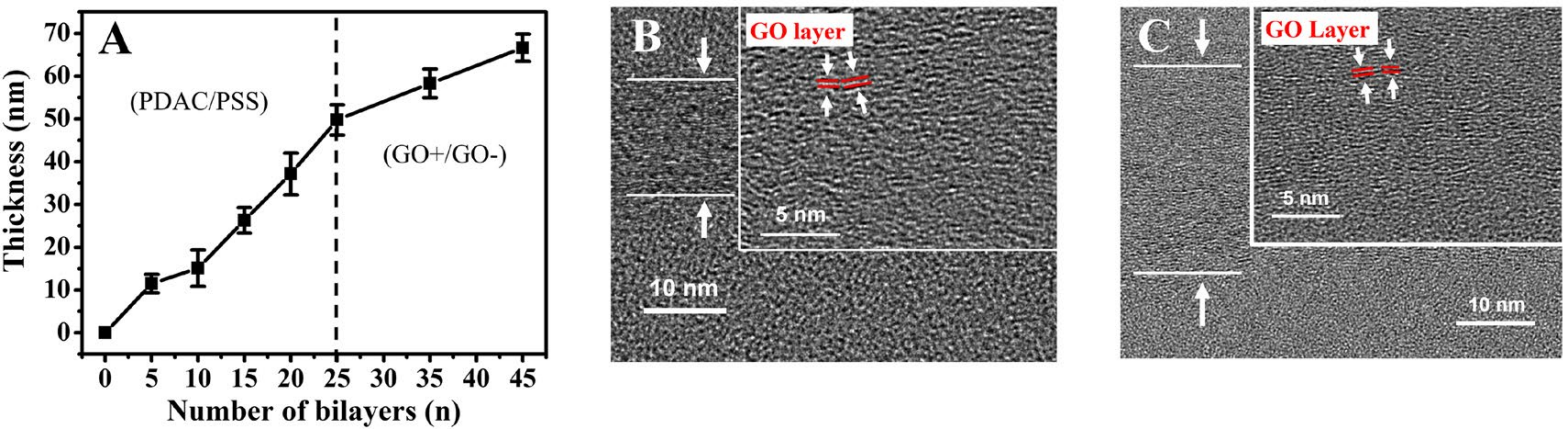
Thickness growth profile and TEM cross-sectional images. (**A**) Thickness growth profile of a spray-assisted LbL-assembled (PDAC/PSS)_n_(GO+/GO−)_n_ film. (**B**) TEM cross-sectional images of (PDAC/PSS)_25_(GO/GO)_20.5_ and (**C**) (PDAC/PSS)_25_(GO/GO)_40.5_. A higher magnification image is shown in the inset, which represents LbL-assembled GO sheets.

The thickness of the (PDAC/PSS) layer was controlled by means of the number of LbL assembly cycles and the salt (NaCl) concentration (Fig. 2 and Fig. S1). The appropriate salt concentration and PDAC/PSS layer thickness were determined by gas permeance analysis. Too much salt exerts an unfavorable effect on the base layer and can lead to gas leakage. At a low salt concentration (0.1 M NaCl), the CO_2_ and N_2_ permeabilities are 105.75 GPU and 3.17 GPU, respectively, with a layer thickness of 60 nm. Conversely, at a high salt concentration (1.0 M NaCl), the CO_2_ and N_2_ permeabilities are 423 GPU and 282 GPU, respectively, even though the layer thickness is over 1 μm. In order to shorten the CO_2_ molecule pathway, the salt concentration was fixed at 0.1 M. Furthermore, the PDAC/PSS layer is not only a defect barrier but also plays an important role in CO_2_ selectivity. The amine group of PDAC facilitates the CO_2_ transport by reversible reaction with CO_2_^30^. The reaction between CO_2_ and the amine group can be explained by the zwitterion reaction mechanism that is proposed by Caplow (1968) and Danckwerts (1979) as shown below^31, 32^:

1 $${{\rm{CO}}}_{2}+{{\rm{R}}}_{2}{\rm{NH}}\leftrightarrow {{\rm{R}}}_{2}{{\rm{NH}}}^{+}{{\rm{COO}}}^{-}$$

2 $${{\rm{R}}}_{2}{{\rm{NH}}}^{+}{{\rm{COO}}}^{-}+{\rm{B}}({{\rm{H}}}_{2}{\rm{O}})\leftrightarrow {{\rm{R}}}_{2}{{\rm{NCOO}}}^{-}+{{\rm{BH}}}^{+}({{\rm{H}}}_{3}{{\rm{O}}}^{+})$$

3 $${{\rm{R}}}_{2}{{\rm{NH}}}^{+}{{\rm{COO}}}^{-}+{\rm{B}}({{\rm{R}}}_{2}{\rm{NH}})\leftrightarrow {{\rm{R}}}_{2}{{\rm{NCOO}}}^{-}+{{\rm{BH}}}^{+}({{\rm{R}}}_{2}{{{\rm{NH}}}_{2}}^{+})$$

4 $${{\rm{R}}}_{2}{{\rm{NCOO}}}^{-}+{{\rm{H}}}_{2}{\rm{O}}\leftrightarrow {{\rm{R}}}_{2}{\rm{NH}}+{{{\rm{HCO}}}_{3}}^{-}$$

R: Functional group, B: Base (H_2_O or amine).

CO_2_ reacts with the primary or secondary amine group to form an intermediate zwitterion. The zwitterion is deprotonated by base (H_2_O or the amine itself) to form a carbamate ion. The carbamate then reacts with H_2_O to form bicarbonate if it is not stable. Under mild pressure, carbamate and bicarbonate diffuse down the membrane pathway^33^.

The thickness of the GO layer was also controlled by repeating cycles of LbL assembly and by controlling the pH (Fig. 2 and Fig. S1). Carboxyl-functionalized GO-COOH solutions are negatively charged over the pH range 3–12. The GO-COOH functionalities are fully ionized at pH 12, and the degree of ionization decreases with decreasing pH. Conversely, the amine groups (GO-NH_2_) are fully protonated at pH 3 and begin to loose protons above pH 6.0^34^. To ensure the CO_2_ pathway, we aligned the GO sheets by adjusting the pH value of both the GO-COOH and GO-NH_2_ solutions. The pH of GO-NH_2_ was adjusted to 4.3 to provide sufficient electrostatic interaction with the GO-COOH layers. The pH of the GO-COOH solution was also adjusted to 4.3. At this pH, the carboxylic groups of GO-COOH show limited electrostatic repulsion, which allows sufficient charge density for LbL assembly and the formation of a densely stacked selective GO layer.

The GO layer is the most important selective layer of our membrane. The polar groups such as amine, carboxyl, and hydroxyl groups on the surface of GO layers enhance the permeance of CO_2_ because of their affinity for CO_2_ molecules. Although CO_2_ is a nonpolar gas, it interacts with polar groups through its quadrupole moment^35^. However, gas molecules cannot permeate the GO sheets directly because of its inherent barrier properties. Therefore, gas molecules must pass through the aligned internal spaces of the GO layer, which leads to an enhanced molecular sieving effect. The kinetic diameters of CO_2_ and N_2_ are 0.33 and 0.36 nm, respectively. Thus, the interlayer spacing between aligned GO sheets should be less than 0.36 nm, and this precondition can be achieved by tuning the interactions between the GO sheets^17^.

The thickness growth curve on a Si wafer is shown in Fig. 2A. The smaller quantity of the adsorbed polyelectrolytes than those produced by the dipping method is a result of spray-assisted LbL deposition. The kinetics of this method are such that the adsorption duration is limited to a few seconds, leading to insufficient interdiffusion during the adsorption and rinsing step. A 30-bilayer (PDAC/PSS) film is only 60.4 nm thick, and a 50-bilayer (GO/GO) film is only 60.9 nm thick (Fig. S1), which demonstrates the advantage of our process in terms of the precise control of thickness and short preparation time.

The FT-IR analysis reveals the presence of polar functionalities, including hydroxyl and amine groups, on the GO sheets (Fig. S2). Furthermore, from the increase in the intensity of the OH peak as the number of GO layer increase, it is confirmed that the (GO/GO) layers are successively adsorbed onto the (PDAC/PSS)_25_ supporting layer. We investigated four different kinds of membranes to determine the roles of the polyelectrolyte and GO layers. The surface morphologies of films prepared by spray-assisted LbL deposition were analyzed by using top-view SEM (Fig. S3). The (PDAC/PSS)_25_ film (Fig. S3B) has a flatter surface than the other films (Fig. S3A,C and D), because the NaCl concentration is only 0.1 M. PDAC and PSS are strong polyelectrolytes and are almost fully charged in 0.1 M NaCl, which results in the flat surface. Furthermore, the flat surface ensures that the subsequent (GO/GO) layer will adsorb as we designed. If the GO solution is applied without the (PDAC/PSS) layer, it penetrates through the pores of the PSf membrane, forming an aggregated structure or a stacking conformation on the rough surfaces of the PSf membrane with a disordered structure. (PDAC/PSS)_25_(GO/GO)_10.5_, (PDAC/PSS)_25_(GO/GO)_20.5_, and (PDAC/PSS)_25_(GO/GO)_40.5_ films exhibit the structure of LbL-assembled GO sheets. As the number of GO layers increases, the relative thickness of GO layers with respect to polyelectrolytes layers also increases, which results in a rough surface with wrinkled GO sheets. The wrinkles in the aggregated GO result from the mechanism of spray-assisted LbL deposition. Spray-assisted LbL deposition limits the adsorption time of GO sheet so that interdiffusion doesn’t sufficiently occur compare to conventional LbL method. Furthermore, rinsing step is also limited in a few seconds leading to aggregated structure^36^.

**Figure 3:**
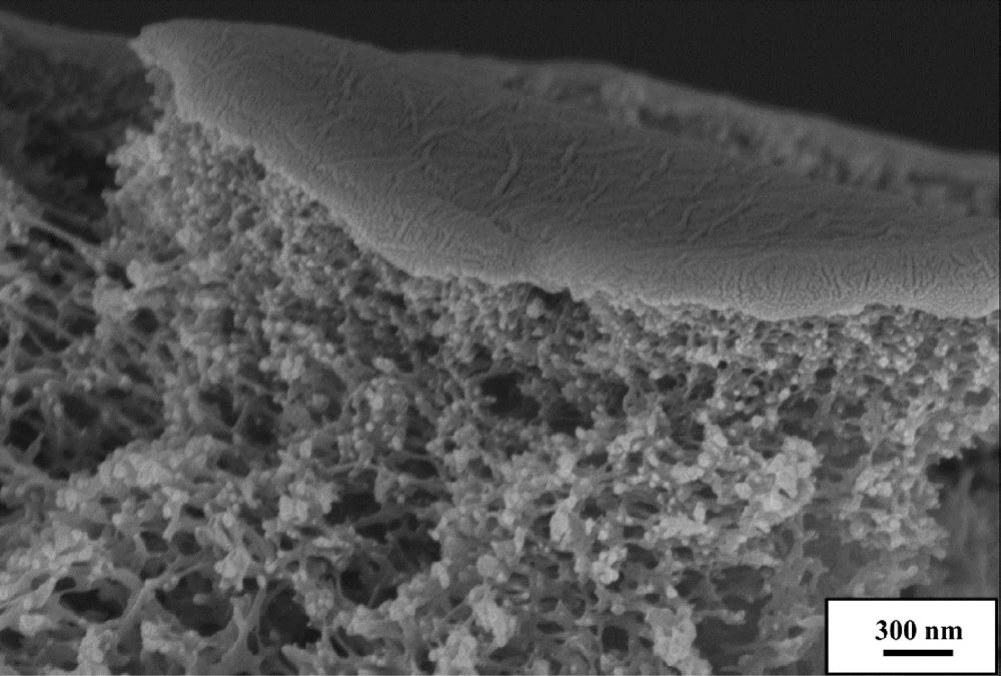
AFM height images of LbL multilayer films deposited on membranes. (A) Bare membrane, (B) (PDAC/PSS)_25.5_, (C) (PDAC/PSS)_25_(GO + GO−)_10.5_, (D) (PDAC/PSS)_25_(GO + GO−)_20.5_, and (E) (PDAC/PSS)_25.5_(GO/GO)_40.5_.

**Figure 4:**
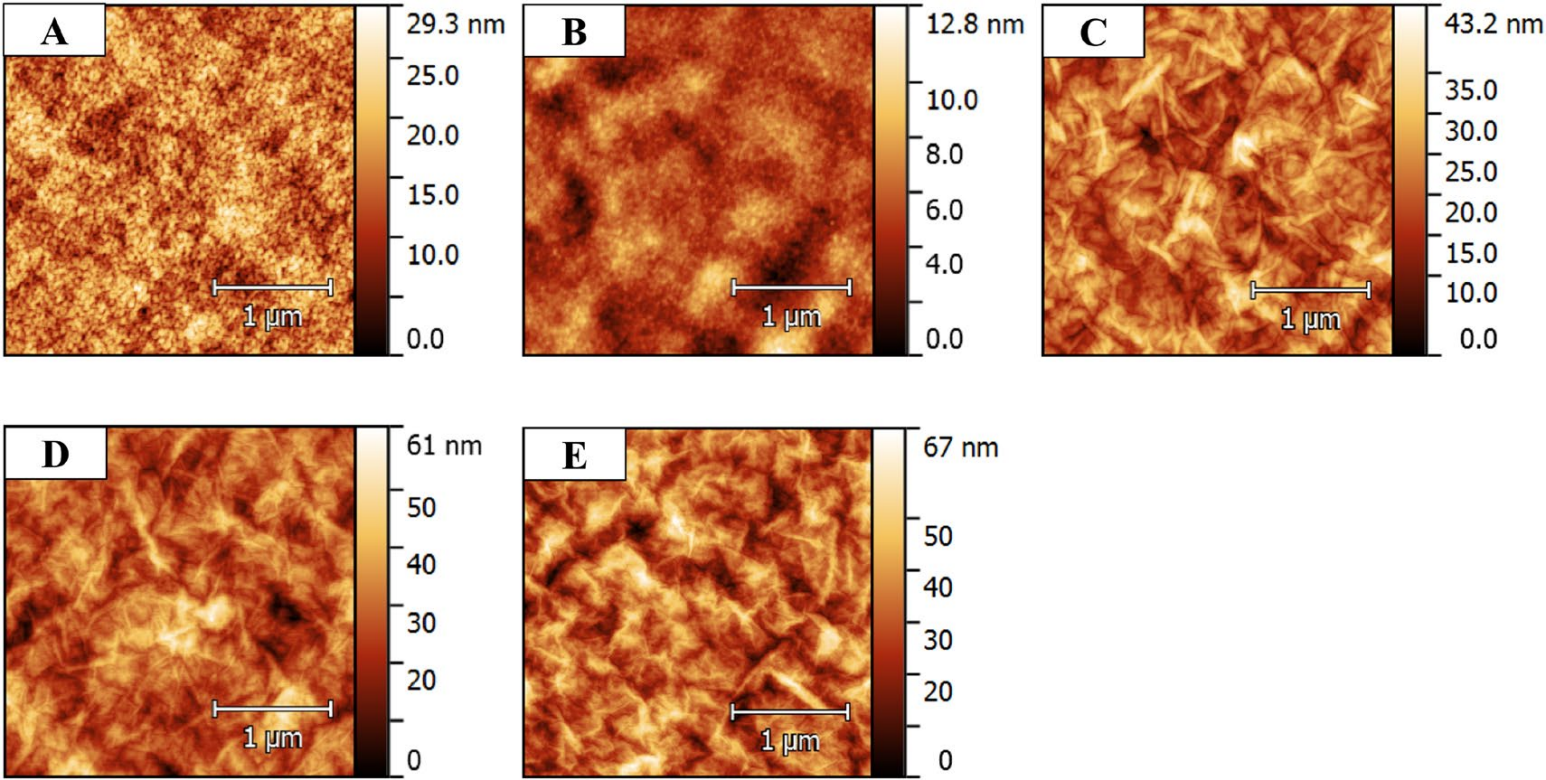
Tilted cross-sectional SEM image. Tilted cross-sectional SEM image of a (PDAC/PSS)_25_(GO/GO)_20.5_ membrane.

The surface morphologies of films were also analyzed by noncontact mode AFM (Fig. 3 and Fig. S4). Figure 3A shows the surface morphology and pore size of a PSf membrane. The roughness of PSf membrane is 4.0 nm RMS (root-mean-square). After deposition of (PDAC/PSS)_25_, the surface becomes smoother (RMS: 1.4 nm), because the membrane surface is fully covered by the polyelectrolyte layer (Fig. 3B). The subsequently adsorbed GO layers are shown in (Fig. 3C–E). The roughness of the layers increases (RMS: 5.4 nm for (PDAC/PSS)_25_(GO/GO)_10.5_, 7.6 nm for (PDAC/PSS)_25_(GO/GO)_20.5_, and 8.3 nm for (PDAC/PSS)_25_(GO/GO)_40.5_), because of the wrinkled structure of the GO sheets. The edges of the graphene oxide sheet are overlapped by other graphene oxide sheets, forming an interlocking jigsaw-type structure with fewer surfaces cracks, and thus ensuring the N_2_ barrier effect of the GO layers (Fig. 4). This interlocking GO sheet-like surface structure may result from the limited electrostatic repulsion of GO sheets. The protonated or deprotonated functional groups (COO^−^ and NH_3_^+^) at the edge of GO sheet (GO-COOH and GO-Amine) have repulsion. However, since pH value of GO solution was adjusted (GO-COOH: 4.3, GO-Amine: 4.3), the repulsion force was limited maintaining enough charge for successful LbL layer formation.

In the cross-sectional TEM images of (PDAC/PSS)_25_(GO/GO)_20.5_ and (PDAC/PSS)_25_(GO/GO)_40.5_ (Fig. 2B,C), the amorphous region at the bottom represents the (PDAC/PSS) layer and the upper laminar region indicates the GO/GO layer. The GO sheets are deposited on the polyelectrolyte layer with a LbL-assembled GO structure, as shown in the inset images. The inset TEM image shows that the LbL-assembled GO sheets are not uniformly stacked. A possible explanation for this structure is that, compared to the pristine regularly stacked graphene sheet, GO is not a perfect two-dimensional material and has many functional groups, which lead to an irregular structure. However, even though the GO sheets are irregularly stacked, there are no significant defects or aggregates in the internal space of the film. The inset image also indicates that the maximum d-spacing of the GO sheets is approximately 0.78 nm. Since d-spacing includes the thickness of the GO, interlayer spacing can be calculated as ca. 0.43 nm. Although the maximum size of the internal space is significantly larger than the size of gas molecules such as CO_2_ (0.33 nm) and N_2_ (0.36 nm), the multilayer structure of the GO/GO layer enables the separation of CO_2_ molecules.

The permeance and CO_2_/N_2_ selectivity of each film is shown in Table 1. The (PDAC/PSS)_25_ membrane was investigated to confirm that CO_2_ transport is facilitated by amine groups in the PDAC layer. The CO_2_/N_2_ selectivity is 3.7, and the CO_2_ permeance is 193.9 GPU. The increased solubility of CO_2_ resulting from its quadrupole moment also enhances selectivity. It was anticipated that the (PDAC/PSS)_25_(GO/GO)_10.5_ membrane would exhibit increased CO_2_/N_2_ selectivity. However, the CO_2_ and N_2_ permeance increase while the CO_2_/N_2_ selectivity decreases slightly. This unexpected result can be explained by the surface coverage and increased surface area of the GO layer. Although GO appears to fully cover the surface (Fig. S3), 10.5 GO bilayers do not block N_2_ sufficiently; this increases N_2_ permeance due to increased surface area. When the GO thickness exceeds 20 bilayers, it effectively blocks N_2_ permeation while retaining permeance to CO_2_ and results in increased CO_2_/N_2_ selectivity. (PDAC/PSS)_25_(GO/GO)_20.5_ and (PDAC/PSS)_25_(GO/GO)_40.5_ achieve selectivities of 11.3 and 15.3, respectively, and CO_2_ permeances of 1269.0 and 1175.0 GPU, respectively. Furthermore, both CO_2_ and N_2_ permeance of membranes increased after GO layer deposition onto the polyelectrolytes layer. This phenomenon can be explained by the change of support polyelectrolytes layer. When GO solution is sprayed onto the polyelectrolytes layer, polyelectrolytes layer is slightly swollen by GO solution. Polyelectrolytes films are sensitive to water and swollen in humid condition^37^. However, since the adsorption time of spray assisted LbL method is limited in a few seconds, the amount of swelling was also limited so that permeance of both CO_2_ and N_2_ gas was increased slightly maintaining selectivity.

**Table 1:** Selectivity and gas permeance of (PDAC/PSS)_25_, (PDAC/PSS)_25_(GO/GO)_10.5_, (PDAC/PSS)_25_(GO/GO)_20.5_, and (PDAC/PSS)_25_(GO/GO)_40.5_ membranes.

Membranes	Permeance (GPU)		Selectivity
	CO_2_	N_2_	CO_2_/N_2_
(PDAC/PSS)_25_	193.88	52.10	3.72
(PDAC/PSS)_25_(GO/GO)_10.5_	1005.01	275.38	3.64
(PDAC/PSS)_25_(GO/GO)_20.5_	1269.00	112.25	11.31
(PDAC/PSS)_25_(GO/GO)_40.5_	1175.03	76.63	15.33

**Figure 5:**
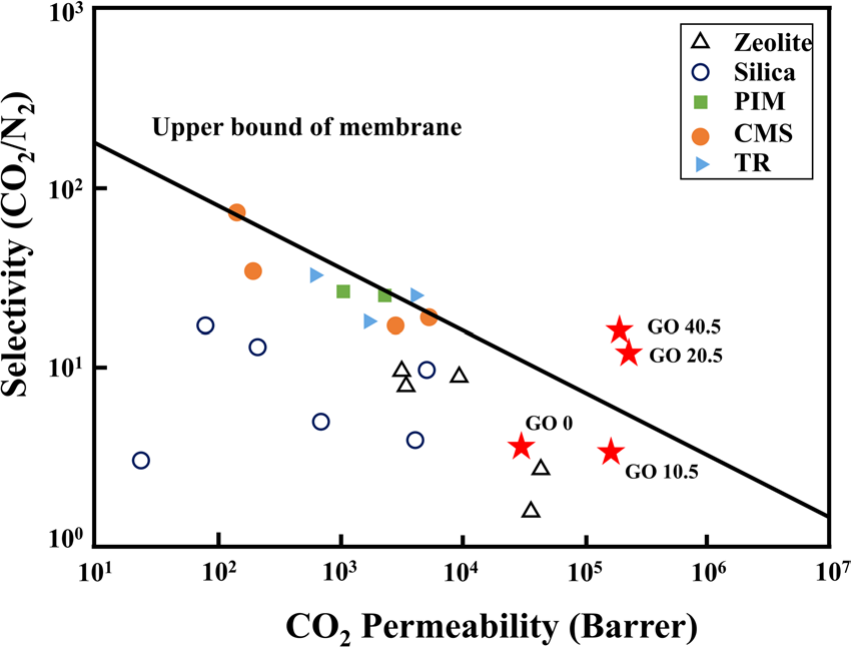
Relationship between CO_2_ permeability and CO_2_/N_2_ selectivity for GO membranes in the dry state. GO 0, GO 10.5, GO 20.5 and GO 40.5 represent (PDAC/PSS)_25.5_, (PDAC/PSS)_25_(GO + GO−)_10.5_, (PDAC/PSS)_25_(GO + GO−)_20.5_, and (PDAC/PSS)_25.5_(GO/GO)_40.5_ respectively.

For CO_2_ separation from a mixed gas, a CO_2_/N_2_ selectivity of greater than 70 and a minimum CO_2_ permeance of 100 Barrels for a membrane thickness of 0.1 μm (a permeance of 1000 GPU) are required for industrial applications^38^. Our (PDAC/PSS)_25_(GO/GO)_20.5_ and (PDAC/PSS)_25_(GO/GO)_40.5_ membranes satisfy this commercial demand (Table 1 and Fig. 5). Our membrane measurements were conducted under dry conditions; however, humidity increases CO_2_/N_2_ selectivity due to facilitated CO_2_ transport.

The CO_2_ permeabilities of the (PDAC/PSS)_25_ and (PDAC/PSS)_25_(GO/GO)_10.5_ membranes are 29403.8 and 152419.8 Barrer, respectively, and the CO_2_/N_2_ selectivities of both are ca. 3.7. This separation efficiency is similar to that of other polymeric membranes, such as carbon molecular sieves (CMS) and polymers of intrinsic microporosity (PIMs). However, the CO_2_ separation performance increases markedly for (PDAC/PSS)_25_(GO/GO)_20.5_ and (PDAC/PSS)_25_(GO/GO)_40.5_, which exhibit CO_2_ permeabilities of 192456.5 and 178205.1 Barrels, respectively, and CO_2_/N_2_ selectivities of 11.3 and 15.3, respectively (Fig. 5). These performances exceed those of other polymeric membranes, including PIMs^39^, thermally rearranged (TR) polymer membranes^40^, silica membranes, and zeolite membranes^41^. The results indicate that spray-assisted LbL-assembled nanofilms have the ability to separate CO_2_, although their thicknesses are less than 100 nm. In addition, the (PDAC/PSS) polyelectrolyte layer possesses CO_2_/N_2_ selectivity because of its amine groups, and the CO_2_ selectivity increases with the number of GO layers on the polyelectrolyte layer.

## Conclusion

In conclusion, we have investigated the ability of structure- and thickness-controlled polyelectrolyte/GO membranes prepared by spray-assisted LbL assembly to separate CO_2_. The molecular sieving effect of aligned GO layers produced by pH control of GO solutions and a high CO_2_ affinity resulting from polar functional groups on GO sheets lead to excellent performance for multilayer films less than 100 nm in thickness. Further research on methods for aligning GO layers in a highly ordered form should enhance membrane performance.

## Materials and Methods

### Materials

Graphite (20 microns), potassium permanganate, potassium persulfate, phosphorus pentoxide, ethylenediamine, poly(diallyldimethylammonium chloride) (MW 20–35 k, PDAC), and poly(sodium-4-styrenesulfonate) (MW 70 k, PSS) were purchased from Sigma Aldrich. 1-(3-Dimethylaminopropyl)-3-ethylcarbodiimide methiodide (EDC) was purchased from Alfa Aesar. Macroporous polysulfone support having average 0.1 *μ*m pore was purchased from Toray Inc., Ltd., Tokyo, Japan.

### Synthesis of GO

GO was synthesized from graphite by a modification of Hummer's method^42, 43^. Negatively charged GO-COOH was prepared by oxidation with acid treatment to introduce carboxylic acid groups. Graphite powder (1 g) was added to a mixture of concentrated H_2_SO_4_ (4 mL), K_2_S_2_O_8_ (0.8 g), and P_2_O_5_ (0.8 g) at 80 °C. The dark mixture was stirred for 4.5 h. Distilled water was slowly added to the mixture, which was filtered and washed until the rinse water reached pH 6.0. After drying overnight at room temperature, the pre-oxidized powder was added to concentrated H_2_SO_4_ (26 mL) at 0 °C. KMnO_4_ was added slowly to maintain the solution temperature below 30 °C. The solution was stirred for 2 h at 36 °C, and distilled water (46 mL) was added gradually to maintain the solution temperature below 60 °C. The solution was stirred for 2 h at 36 °C. The reaction was terminated by addition of distilled water (140 mL) and 30% H_2_O_2_ solution (2.5 mL). The solution was filtered and rinsed with 10% HCl solution (350 mL). Dialysis was performed to remove the residual ions and HCl solution.

### Preparation of polyelectrolyte and GO solutions

PDAC and PSS were dissolved in aqueous 0.1 M NaCl at 1 mg/mL each. Negatively charged GO-COOH solution was prepared by dissolving GO powder in pH 4.3 DI water at a concentration of 0.5 mg/mL with ultrasonication. The average flake size of GO was about 850 nm with wide size distribution from hundreds of nanometers to a few micron meters. We used as synthesized without further size separation or purification of GO solution. A positively charged GO-NH_2_ solution was prepared by functionalizing amine groups on the edge of negatively charged GO-COOH by an EDC-mediated reaction between excess ethylenediamine and carboxylic acid groups^44^. Excess reactants and byproducts were removed by dialysis for one week. The pH values of the GO-COOH and GO-NH_2_ solutions were adjusted to 4.3 to provide an ionic strength of more than 50%. Zeta-potential of GO-COOH solution was −53.83 mV and GO-NH_2_ was +57.77 mV.

### Spray-assisted LbL film deposition on a silicon wafer and PSf membrane

Multilayer films were constructed on a 2.5 cm × 2.5 cm Si wafer and a 1.5 cm diameter circle of PSf membrane. Substrates were thoroughly cleaned in piranha solution (sulfuric acid/hydrogen peroxide 75/25 v/v) for 5 min following O_2_ plasma treatment (Femto Science) to produce a negatively charged surface. The substrates were then fixed on a film holder for spray-assisted LbL deposition. PDAC solutions (0.1 M NaCl) were sprayed four times to cover the entire substrate surfaces. Next, distilled water was sprayed four times to remove weakly attached polyelectrolytes. This half step was repeated with the PSS solutions (0.1 M NaCl), and the complete step was repeated to obtain the desired number of PDAC/PSS bilayers. GO-NH_2_/GO-COOH layers were fabricated by the same procedure.

### Characterization

The thicknesses of the multilayer thin films on the Si wafers were measured by profilometry (Dektak 150, Veeco) and field-emission scanning electron microscopy (FE-SEM, SIGMA, Carl Zeiss). RMS roughness values and surface morphologies of membranes were obtained by atomic force microscopy (AFM, NX-10, Park Systems). The presence of functional groups in multilayer films was verified by Fourier-transform infrared spectroscopy (FT-IR-4700). The permeance of GO membranes was measured with a bubble meter to determine the value at a given membrane area and pressure. Permeance, defined as pressure-normalized mass flux, is expressed mathematically as J = Q × Δp, where Q is the permeance, J is the mass flux, and Δp is the pressure difference across the GO membrane. The volume of the permeating gas determines the permeance. Selectivity is defined as the ratio of the CO_2_ and N_2_ permeabilities. Gas permeance is expressed in terms of gas permeation units (GPU), where 1 GPU = 1 × 10^−6^ cm^3^ (STP)/(cm^2^ s cm-Hg).

## Electronic supplementary material

Supporting Info
