# Supplementary material for: Highly Permeable Graphene Oxide/Polyelectrolytes Hybrid Thin Films for Enhanced CO2/N2 Separation Performance
Source: Sci Rep. 2017 Mar 28;7:456. doi: 10.1038/s41598-017-00433-z (PMC5428404; doi:10.1038/s41598-017-00433-z)
Supplement: Supplementary file 1 — Supporting Info [file 41598_2017_433_MOESM1_ESM.pdf]

Supporting Information Figures

Highly Permeable Graphene Oxide/Polyelectrolytes Hybrid Thin Films for Enhanced CO_2/N_2 Separation Performance

Jiwoong Heo[1], Moonhyun Choi[1], Jungyun Chang[2], Dahye Ji[2], Sang Wook Kang[2,*] and Jinkee Hong[1,*]

[1]Department of Chemical Engineering & Material Science, Chung-Ang University, 47 Heukseok-ro, Dongjak-gu, Seoul 156-756, Republic of Korea.

[2]Department of Chemistry, Sangmyung University, Seoul 110-743, Republic of Korea.

*Corresponding author: E-mail address: jkhong@cau.ac.kr, Tel.: +82-2-820-5561, swkang@smu.ac.kr, Tel.: +82-2-781-7601

Supporting Information Figure S1

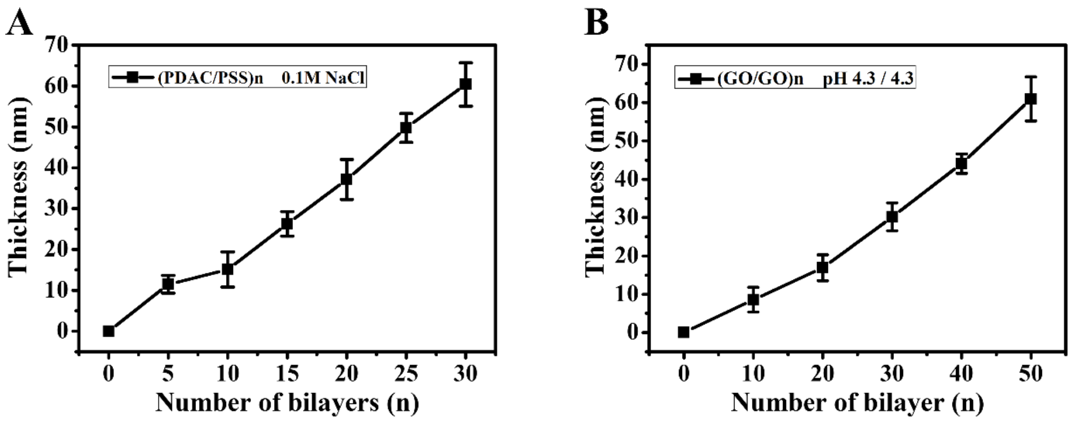

Figure S1. Thickness growth profiles of spray-assisted LbL assembled films deposited on a Si wafer. (A) $(PDAC/PSS)_n$ and (B) $(GO/GO)_n$ films.

The thickness growth profiles of the $(PDAC/PSS)_n$ and $(GO/GO)_n$ were measured individually. Both multilayer film showed that linear increase in thickness as a function of the number of bilayers. Average increase of $(PDAC/PSS)_n$ and $(GO/GO)_n$ film was 1.98 nm per bilayer and 1.22 nm per bilayer respectively. The lesser increase of thickness than those of dipping LbL process is result from kinetic of spray-assisted LbL deposition.

Supporting Information Figure S2

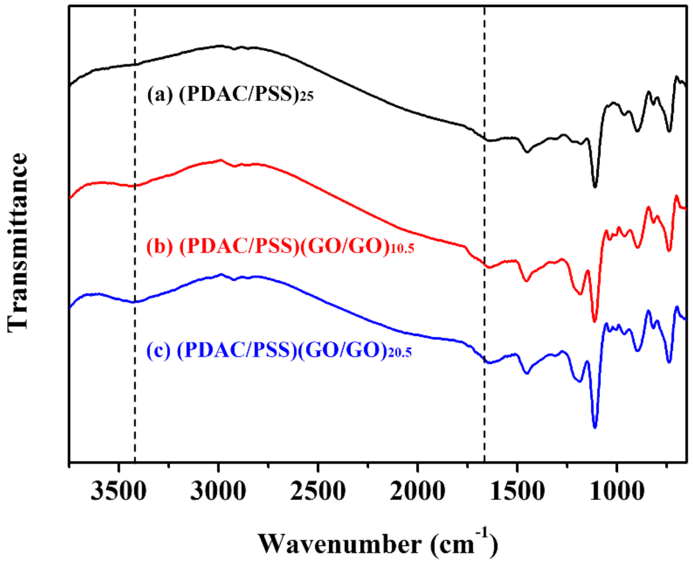

Figure S2. FT-IR spectra of (a) (PDAC/PSS)$_{25}$, (b) (PDAC/PSS)$_{25}$(GO/GO)$_{10.5}$, and (c) PDAC/PSS)$_{25}$(GO/GO)$_{20.5}$.

We analyzed adsorption of (GO/GO)$_n$ film on the (PDAC/PSS)$_{25}$ base layer by ATR mode of FT-IR. Hydroxyl groups and amine groups on the GO sheets were observed as a broad peak at 3450 and 1620. These peaks were gradually increased as the number of bilayer increased, which represented GO sheets were stacked on the base layer.

Supporting Information Figure S3

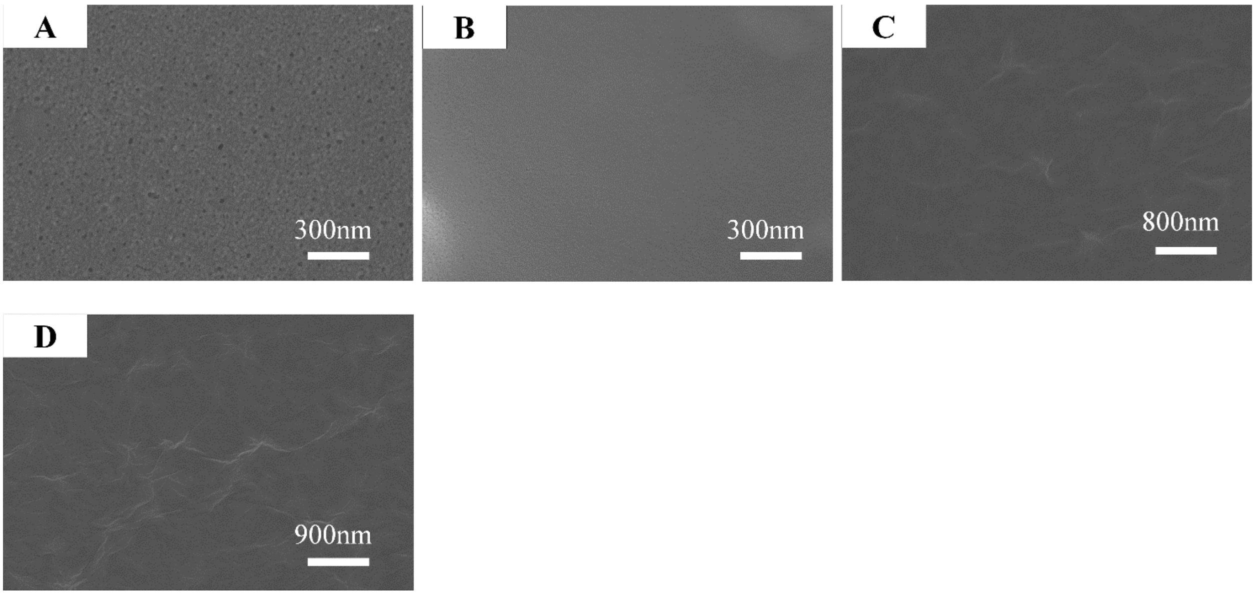

Figure S3. SEM images of LbL multilayer films deposited on membranes. (A) Bare membrane, (B) $(PDAC/PSS)_{25.5}$, (C) $(PDAC/PSS)_{25}(GO/GO)_{10.5}$, and (D) $(PDAC/PSS)_{25}(GO/GO)_{20.5}$.

The surface morphologies of membranes were measured by top-view SEM. Pores of bare PSf membrane were covered by $(PDAC/PSS)_{25.5}$ film, which has a flatter surface compare to other surfaces. As the number of GO layer increases, the ratio of wrinkled GO structure also increased, which result in rough surface.

Supporting Information Figure S4

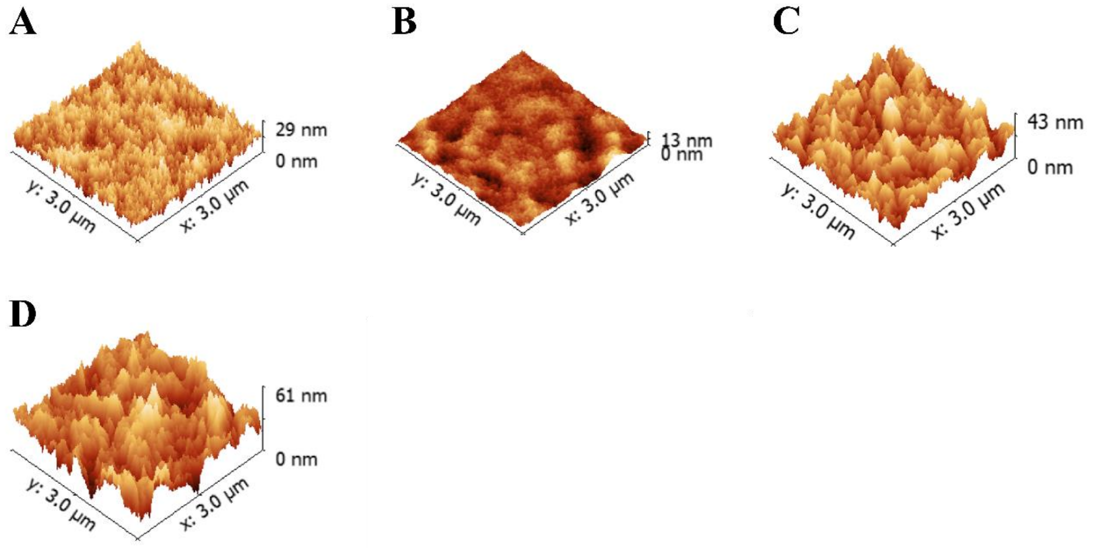

Figure S4. 3D view of AFM images of LbL multilayer films deposited on membranes. (A) Bare membrane, (B) $(PDAC/PSS)_{25.5}$, (C) $(PDAC/PSS)_{25}(GO/GO)_{10.5}$, and (D) $(PDAC/PSS)_{25}(GO/GO)_{20.5}$.

The surface roughness of membranes was investigated by 3D view of AFM. Comparatively rough surface of PSf membrane was flattened by $(PDAC/PSS)_{25.5}$ film. Surface roughness gradually increased as the number of $(GO/GO)_n$ layer increased, which result from wrinkle structure of GO sheets.
